# Fabrication and Characterization of Biopolymers Using Polyvinyl Alcohol and Cardanol-Based Polyols

**DOI:** 10.3390/molecules29204807

**Published:** 2024-10-11

**Authors:** Da Hae Lee, Yun Ha Song, Hee Ju Ahn, Jaekyoung Lee, Hee Chul Woo

**Affiliations:** Department of Chemical Engineering, Pukyong National University, 45 Yongso-ro, Nam-gu, Busan 48513, Republic of Korea; ekgo2364@pukyong.ac.kr (D.H.L.); yhsong27@pukyong.ac.kr (Y.H.S.); duksikahn@hanmail.net (H.J.A.)

**Keywords:** biopolymer, cardanol, polyvinyl alcohol, biomass, crosslinking, swelling

## Abstract

Biodegradable polymers are getting attention as renewable alternatives to petroleum-based plastics due to their environmental benefits. However, improving their physical properties remains challenging. In this work, biodegradable biopolymers (PVA-PCD) were fabricated by chemically crosslinking petroleum-based polyvinyl alcohol (PVA) with biomass-derived cardanol-based polyols (PCD). Biopolymers were characterized using various techniques, including Fourier-transform infrared (FT-IR), X-ray diffraction (XRD), thermogravimetric analysis (TGA), and swelling tests. Cardanol, the raw material, was converted into polyols via epoxidation followed by hydroxylation. FT-IR analysis confirmed that PVA-PCD biopolymers were crosslinked between the hydroxyl groups of PVA and PCD and the aldehydes of crosslinker glutaraldehyde (GLU), accompanied by the formation of acetal groups with ether bridges. XRD showed that the crystallinity of crosslinked polymers decreased, indicating that crosslinking occurs disorderly. TGA exhibited that GLU significantly improved the thermal stabilities of PVA and PCD-PVA polymers, as evidenced by increased decomposition temperatures. On the other hand, the effect of PVA/PCD ratios was minor on biopolymers’ thermal stabilities. Swelling tests revealed that increased crosslinking density decreased the swelling ratio, suggesting that PVA-PCD biopolymers become more hydrophobic with high brittleness, high strength, and low swelling capacity. In summary, this study demonstrates that PVA-PCD biopolymers fabricated from biomass-derived materials have potential for various applications, such as biodegradable materials and sustainable packaging.

## 1. Introduction

Currently, plastics derived from petroleum resources have been widely used in various applications, such as packaging, containers, and films, due to their excellent physicochemical properties, including durability, heat resistance, light weight, and low cost. As a result, their consumption continues to increase [1]. However, the problem of how to dispose and manage the increasing waste plastics is a very challenging and a critical environmental issue because these plastics are difficult to decompose. It is reported that approximately 8.3 billion tons of plastic have been produced globally from 1950 to 2015, with ~60% of the total amounts being discarded, landfilled, or released into the environment. However, the recycling rates remain low, at only ~7.2%, and the amount of waste plastics are expected to reach ~26 billion tons by 2050 [1].

Petroleum-based plastics are non-biodegradable and require decades to centuries to decompose naturally. To address the issue of non-biodegradable plastic wastes, various methods, such as incineration, landfilling, recycling, or energy recovery, are used. In the view of sustainable usage of plastics, recycling and energy recovery are preferred for resource circulation and energy conservation. However, due to high costs, most plastic wastes are still incinerated or landfilled [2]. The incineration process releases greenhouse gases, such as carbon dioxide, and various toxic substances, contributing to environmental pollution. Plastics that are landfilled or discharged into marine environments persist for a long time, causing severe environmental contamination and ecological disruption. Therefore, the use of petroleum-based, non-biodegradable plastics is increasingly restricted due to challenges in waste management and harmful environmental issues. In response, global efforts are underway to reduce plastic consumption and seek alternatives that mitigate these environmental impacts [3,4].

Among the various efforts to address the plastic waste crisis, biodegradable plastics are getting significant attention as a potential alternative to non-biodegradable plastics. Biodegradable plastics are materials that can be broken down into water and carbon dioxide by living organisms, such as bacteria or microorganisms. Biodegradable plastics can be broadly classified into the following two categories: petroleum-based and bio-based materials. Petroleum-based biodegradable materials include polyvinyl alcohol (PVA), polybutylene succinate (PBS), and polybutylene adipate terephthalate (PBAT). Although these materials exhibit excellent physical properties such as heat resistance and light weight, they are not the right tools to accomplish the sustainable utilization of plastics to solve environmental issues. On the other hand, bio-based materials include polylactic acid (PLA), thermoplastic starch (TPS), and polyhydroxyalkanoates (PHA), which are derived from rapidly renewable resources, such as sugarcane, starch, and corn. Bio-based materials are attractive as sustainable materials that contribute to carbon reduction and the corresponding market demands for eco-friendly products [5]. Natural polymers, such as starch, chitin (extracted from crustacean shells), and cellulose (derived from lignocellulosic biomass), are highly promising as raw materials for biodegradable plastics due to their biocompatibility and environmental friendliness. However, their mechanical properties, such as strength and processability, remain inferior to those of conventional petroleum-based plastics, limiting their potential as direct substitutes. In particular, packaging for food or cosmetics should be eco-friendly and biocompatible, with the proper mechanical strengths and chemical stabilities. Consequently, the research and development of new biodegradable plastics with improved physical properties and processing techniques is essential to minimize the environmental impact of the rapidly expanding plastics market [5,6,7,8,9].

Making composite materials is one of the widely adopted strategies to maintain the advantages and compensate for the disadvantages of each component material. For example, cardanol, which is a bio-based polyol derived from cashew nut shell liquid, has been used to enhance the mechanical properties of polyurethane films. Bio-polyurethane films produced with cardanol-derived polyols exhibit superior tensile strength and thermal stability compared to conventional polyurethane films. Furthermore, antibacterial tests using Escherichia coli have demonstrated that these films achieve a 99.9% reduction in bacterial growth, confirming the antimicrobial effect imparted by the addition of cardanol-derived polyols [10,11,12,13]. Thus, cardanol-derived bio-polyols are suitable to enhance mechanical properties and impart antimicrobial characteristics to bio-based biodegradable plastics. Various parameters, such as the ratio of each component and the synthesis protocols, can affect the polymer composite materials; however, how each variable can affect the properties of biopolymer composites is not well understood. In particular, the details of cardanol-based polyols-derived composite materials are limited.

In this study, we aim to develop a polyvinyl alcohol (PVA)-based biodegradable film by incorporating a bio-polyol derived from cardanol (PCD), which is known for its antimicrobial properties. We fabricated PVA-PCD biopolymers by controlling the ratio of PVA/PCD and performed multiple characterizations to investigate its chemical structures and thermal stabilities. Furthermore, we evaluated PVA-PCD biopolymers using swelling tests to study the potential applicability of these biodegradable biopolymers in various practical applications.

## 2. Results and Discussions

### 2.1. FT-IR

**Figure 1:**
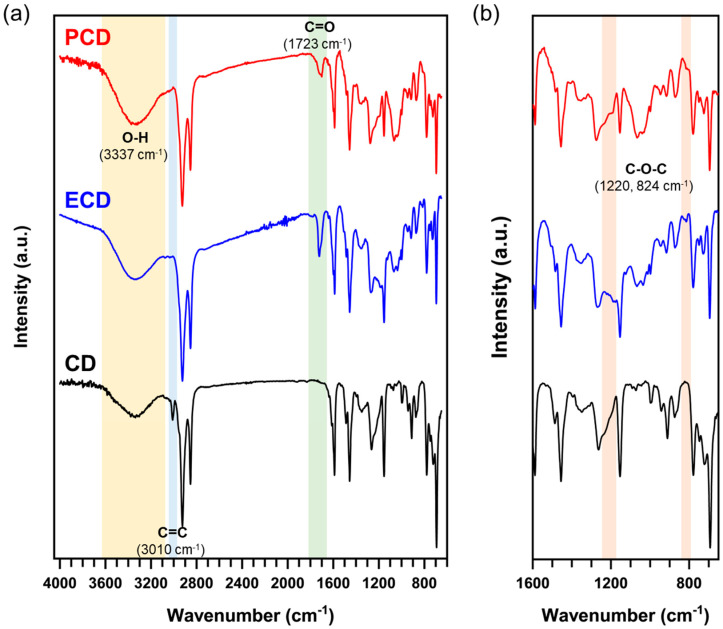
FT−IR spectra of CD, ECD, and PCD (**a**) full spectrum, (**b**) range of 650–1450 cm^−1^.

Cardanol (CD), the raw material for biopolymer in this study, was converted to epoxidized cardanol (ECD), and polyols derived from cardanol (PCD) and the chemical structural changes were analyzed using FT-IR spectroscopy, as shown in Figure 1, with detailed information on the functional groups provided in Table S1.

In the cardanol sample (CD), the O-H in-plane bending vibration of the phenolic group appeared as a broad peak at 1350 cm^−1^, and an O-H absorption peak due to hydrogen bonding was observed at 3339 cm^−1^. The C=C stretching vibrations of the aromatic ring appeared at 1589, 1487, and 1456 cm^−1^, while the C-H out-of-plane bending vibrations of the aromatic ring were observed at 692 and 773 cm^−1^. Regarding the unsaturated hydrocarbons in the alkyl chain, C-H stretching vibrations were observed at 3010 cm^−1^, and C=C stretching vibrations appeared at 1611 cm^−1^. The alkyl chain attached to the benzene ring in the cardanol structure revealed strong symmetric and asymmetric stretching vibrations of methylene and methyl groups around 2853 and 2925 cm^−1^, respectively. Additionally, absorption peaks at 994, 911, and 692 cm^−1^ represent the out-of-plane bending vibrations of the C-H bonds. Among these, the absorption peak at 692 cm^−1^ exhibits a very strong intensity due to the overlap with the C-H bond vibrations of the benzene ring. Furthermore, the absorption peak at 722 cm^−1^ indicates the bending vibrations caused by the rocking of methylene groups within the long alkyl chain, and the peak at 1263 cm^−1^ is attributed to the C-O stretching vibration between the hydroxyl (O-H) group of the phenol and the benzene ring [14,15,16,17,18,19,20]. An FT-IR analysis confirmed that the cardanol (CD) exhibited characteristic peaks consistent with the reported literature, indicating that the sample has been well purified.

Next, epoxidized cardanol (ECD) was compared with the characteristic peaks of the cardanol (CD), as shown in Figure 1. The peaks at 3010 and 1611 cm^−1^, which were associated with the C=C double bonds in the unsaturated alkyl chain, disappeared. Additionally, the intensity of the overlapping absorption peak at 911.2 cm^−1^, which was related to the methylene groups, was significantly reduced. These changes indicate that the C=C bonds were consumed in the epoxidation reaction. Also, a new peak appeared at 824 cm^−1^, corresponding to the C-O-C epoxy group, and the peak of the epoxide ring appeared at 1222 cm^−1^. The new peak at 1723 cm^−1^ represents the C=O carbonyl bond, which is attributed to byproducts, such as acetyl or formyl groups, formed through side reactions between epoxy groups with H^+^, residual H_2_O_2_, or H_2_O. Newly observed peaks at 1035 and 1067 cm^−1^ indicate the band formed by the C-O bond associated with hydroxyl ends-by-side reactions between the epoxy group and water [19,21,22,23,24,25]. In summary, via the epoxidation of cardanol with H_2_O_2_ and acidic conditions, we confirmed that cardanol (CD) was epoxidized by forming epoxy groups at the double bond positions of the alkyl chain in cardanol.

**Scheme 1:**
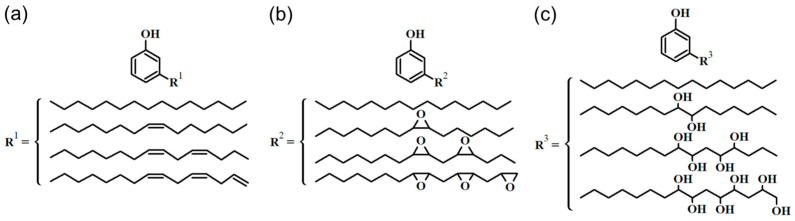
Molecular structure of (**a**) cardanol (CD), (**b**) epoxidized cardanol (ECD), (**c**) polyols derived from cardanol (PCD).

Next, polyols derived from cardanol (PCD) were synthesized from the epoxidized cardanol (ECD), and the FT-IR analysis results are shown in Figure 1. The peaks at 824 cm^−1^ and 1222 cm^−1^, which represent the epoxy group, almost disappeared, and the peak at 1723 cm^−1^, representing the carbonyl group formed as a side reaction, significantly decreased. These results indicate that a substantial portion of these functional groups were successfully converted into polyols. Additionally, the absorption peak at 3337 cm^−1^, which indicates the hydrogen bonding of hydroxyl groups, significantly increased, along with an increase in the intensity of the hydroxyl group peaks at 1035 cm^−1^ and 1067 cm^−1^ [14,18,25]. This result confirms that the epoxy groups in epoxidized cardanol reacted with water under acidic conditions to successfully convert to polyols. Overall, the double bonds in the alkyl chain of the raw cardanol material were converted to polyols derived from cardanol (PCD)-containing hydroxyl groups, and representative structures of each raw material (CD, ECD, and PCD) are summarized in Scheme 1.

We fabricated biopolymers using polyols derived from cardanol. Before testing PCD-mixed biopolymer, we first fabricated the petrochemical-based polymers, using polyvinyl alcohol polymer (PVA) as a reference. The PVA polymer was crosslinked using glutaraldehyde (GLU) as a crosslinker. The GLU was added in amounts ranging from 1 to 30 wt% relative to the weight of PVA and synthesized under acid-catalyzed conditions.

**Figure 2:**
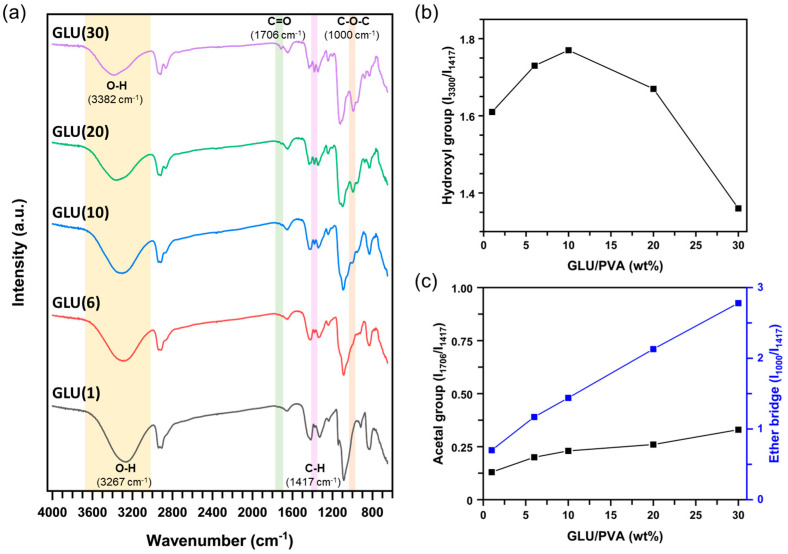
(**a**) FT−IR spectra of crosslinked PVA polymers, ratio GLU/PVA associated with (**b**) hydroxyl group (I_3300_/I_1417_), (**c**) acetal group (I_1706_/I_1417_), and ether bridge (I_1000_/I_1417_).

Structural changes in the crosslinked PVA polymer with changes in GLU amounts were analyzed using FT-IR, with major peaks indicated in Figure 2a. The intensity changes in functional groups after crosslinking were shown, with the relative intensity ratio based on the normalization of the C-H bond peak at 1417 cm^−1^ in the PVA chains, which remain constant irrespective of crosslinking. Figure 2b describes the relative intensity ratio for hydroxyl groups at 3300 cm^−1^, and Figure 2c describes carbonyl groups at 1706 cm^−1^ and ether bridges at 1000 cm^−1^, respectively [26]. Detailed data on the functional groups can be found in Table S2.

**Scheme 2:**
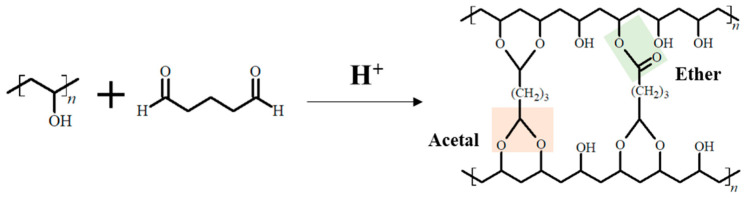
Chemical crosslinking of poly(vinyl) alcohol (PVA) and glutaraldehyde (GLU).

In Figure 2a, the peak at 3200–3400 cm^−1^ corresponds to the hydroxyl group (O-H). The absorption peaks at 2933–2939 cm^−1^ and 1417 cm^−1^ correspond to the asymmetric stretching vibrations of C-H bonds in methylene groups and the C-H stretching vibrations in alkyl chains, respectively. These peaks are typical characteristics of the PVA structure [26,27,28]. As the amount of crosslinker (GLU) increased, the maximum O-H peak shifted from 3267 cm^−1^ to 3382 cm^−1^, with a decrease in intensity, indicating that the hydroxyl groups are consumed in the crosslinking reaction with the aldehyde groups of the crosslinker. The crosslinking process of PVA and GLU is illustrated in Scheme 2. Figure 2b shows the intensity change trends of OH peaks. The I_3300_/I_1417_ ratio increased slightly from 1.61 at 1 wt% GLU to 1.76 at 10 wt% GLU, indicating that some of the GLU was converted to alcohol under acidic conditions. However, after that, up to 30 wt% GLU, the I_3300_/I_1417_ ratio significantly decreased to 1.36, indicating that crosslinking between PVA and GLU occurred well.

It is known that when glutaraldehyde (GLU) is used as a crosslinker, crosslinking can occur either through incomplete crosslinking, in which only some of the four aldehyde groups in GLU participate, or through complete crosslinking, in which all of the aldehyde groups are involved [29,30]. In the case of incomplete crosslinking, the hemiacetal C=O carbonyl group is formed where the related peak is observed at 1706 cm^−1^. On the other hand, complete crosslinking leads to a C-O-C ether bridge formed from the acetal group, whose characteristic peaks are observed at 1000 cm^−1^. These crosslinking processes of PVA and GLU are illustrated in Scheme 2. When the hemiacetal peak intensity by incomplete crosslinking was analyzed with increasing GLU content (Figure 2c), the I_1706_/I_1417_ ratio gradually increased from 0.13 to 0.33. However, the peak at 1000 cm^−1^ of the C-O-C ether bridge by complete crosslinking increased from 0.7 to 2.78 with increasing GLU content, indicating a steeper increase compared to the hemiacetal peak. Therefore, as the crosslinker content increased, crosslinking in PVA occurred more extensively, with a greater proportion of complete crosslinking than incomplete crosslinking.

**Figure 3:**
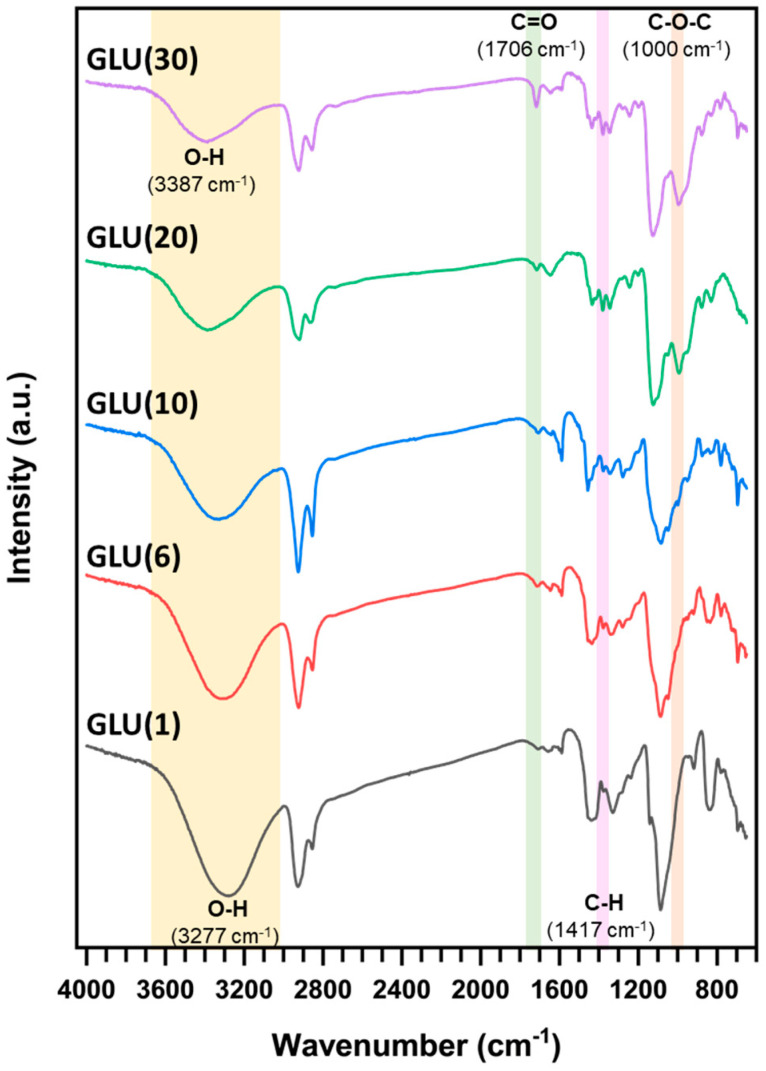
FT-IR spectra of crosslinked PVA(60)-PCD(40) biopolymers.

**Figure 4:**
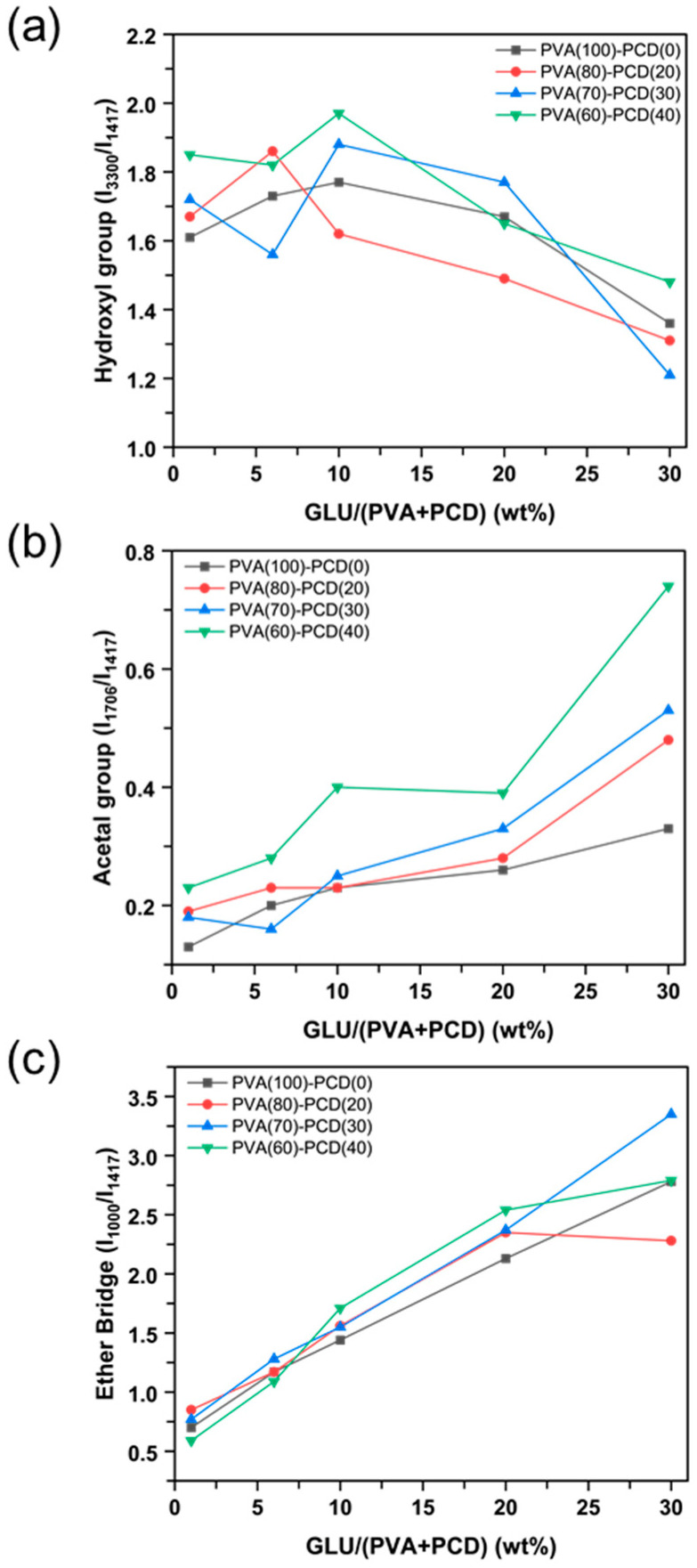
Ratio GLU/(PVA + PCD) associated with (**a**) hydroxyl group (I_3300_/I_1417_), (**b**) acetal group (I_1706_/I_1417_), and (**c**) ether bridge (I_1000_/I_1417_).

We aimed to evaluate the potential application of the PVA-PCD blend polymer as a biopolymer by incorporating and crosslinking PCD with PVA. We controlled PCD contents of 20–40 wt% and GLU contents of 1–30 wt% of (PVA+PCD) and performed an FT-IR analysis to evaluate the degree of crosslinking and chemical structural changes. The FT-IR spectra of the PVA(60)-PCD(40) sample are shown in Figure 3, with major peaks highlighted, and the spectra of the PVA(80)-PCD(20) and PVA(70)-PCD(30) samples are presented in Figure S1a,b, respectively. Detailed data on the functional groups can be found in Table S2. To study the changes in the hydroxyl group (3300 cm^−1^), hemiacetal group (1706 cm^−1^), and ether bridges (1000 cm^−1^), the intensity changes in functional groups relative to the 1417 cm^−1^ peak are summarized in Figure 4.

In Figure 3 and Figure S1, the O-H peak at 3200–3400 cm^−1^ was observed on all samples. Multiple characteristic peaks for the methylene-derived C-H stretching vibrations, associated with alkyl chains present in both PVA and PCD, were detected in the range of 2850–2935 cm^−1^. The C-H bending vibrations in the benzene ring were identified at 695, 750, and 880 cm^−1^, indicating the incorporation of cardanol-derived polyols (PCD), while the bending vibrations from PVA were observed at 780 and 830 cm^−1^. With an increase in the crosslinker content, a shift to higher wavenumbers was noted in the O-H peak, as follows: from 3269 cm^−1^ to 3395 cm^−1^ for PVA(80)-PCD(20), from 3275 cm^−1^ to 3395 cm^−1^ for PVA(70)-PCD(30), and from 3277 cm^−1^ to 3387 cm^−1^ for PVA(60)-PCD(40), along with a decrease in intensity [22,26,27,31,32]. In Figure 4a, I_3300_/I_1417_ did not show the obvious trends under the amounts of GLU <10 wt%, but apparently decreased with the GLU content >10 wt%. This suggests that the crosslinking between PVA and PCD polyols became effective at GLU contents above 10 wt%. The degree of crosslinking was highest in PVA(70)-PCD(30), with 30 wt% GLU. In Figure 4b,c, the hemiacetal peak at 1706 cm^−1^ significantly increased with increasing PCD and GLU contents. This indicates that the formation of hemiacetal groups occurred effectively when PCD was added, compared to PVA only. In other words, the polyols, which are much smaller molecules, are added to the PVA matrix, which is composed of much larger molecules of ~90,000 molecular weights, thus improving the dispersion and promoting the crosslinking between PVA and PCD polyol. The acetal peak at 1000 cm^−1^ also increased with higher PCD content compared to PVA only, but the trend was less pronounced compared to that of the hemiacetal peak. These results suggest that PCD induces a structural hindrance that promotes incomplete crosslinking rather than complete crosslinking.

**Scheme 3:**
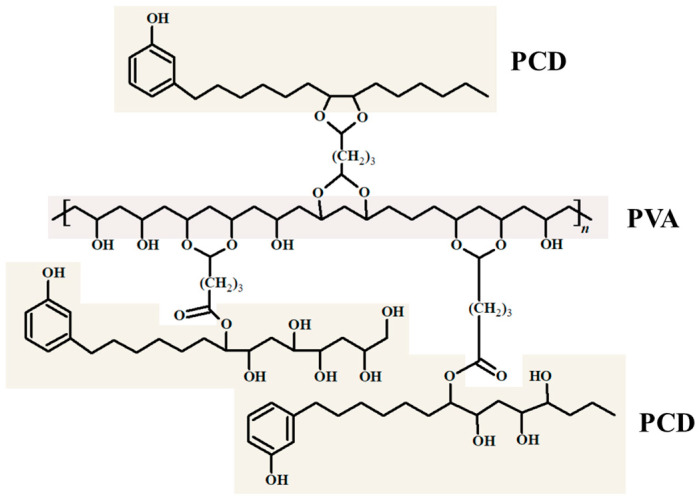
Chemical crosslinking of poly(vinyl) alcohol (PVA), polyols derived from cardanol (PCD), and glutaraldehyde (GLU).

In summary, a synergistic effect in the crosslinking reaction was observed when the PCD-PVA biopolymer was fabricated by adjusting the PCD content to 20–40 wt% and the GLU content to 1–30 wt%. Regardless of the PCD content, an increase in the GLU content led to a reduction in hydroxyl groups, along with an increase in peaks associated with crosslinking, such as acetal and ether bridges (Figure S2). These results confirm that effective crosslinking also occurred with the addition of PCD for the fabrication of the PVA-PCD biopolymer. The relatively small PCD polyol molecules, compared to the large PVA molecules, are believed to disperse well into the spaces between PVA molecules, thereby participating in crosslinking. This trend is similar to the effect of the GLU in the fabrication of PVA polymers alone. It is likely attributed to the similarity in hydroxyl groups attached to the alkyl chains present in both PVA and PCD polyol. The crosslinking mechanism of the PVA-PCD biopolymer is illustrated in Scheme 3.

### 2.2. XRD

**Figure 5:**
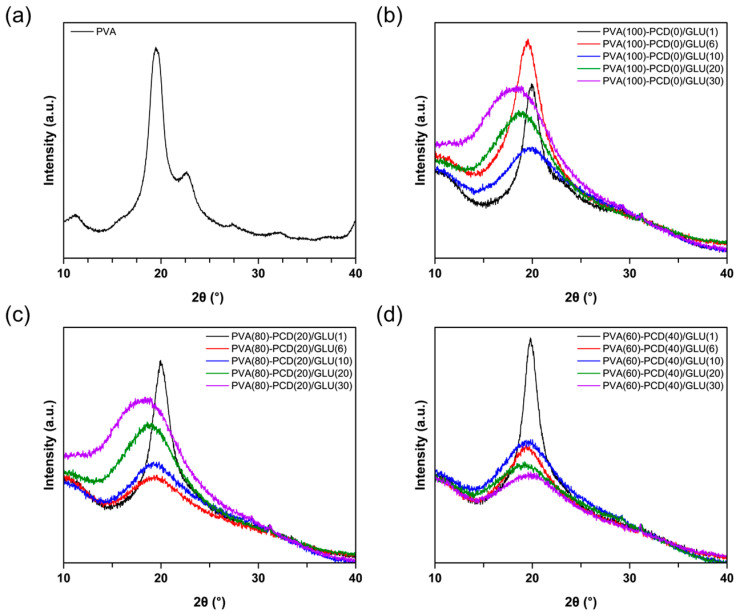
XRD patterns of (**a**) uncrosslinked PVA, (**b**) PVA polymers, (**c**) PVA(80)-PCD(20) biopolymers, and (**d**) PVA(60)-PCD(40) biopolymers.

The crystallinity of the fabricated PVA polymers and PVA-PCD biopolymers was investigated using an XRD analysis, as shown in Figure 5. Figure 5a displays the XRD pattern of the uncrosslinked PVA raw material. The major peaks of the raw PVA polymer were observed at 2θ values of 19.5°, 22.5°, and 40.8°, corresponding to the (101), (200), and (111) planes, respectively, which is consistent with previous studies [30,32,33]. The crystallinity index (CI), which was calculated based on the (101) peak at 2θ = 19.5°, was 75.7%.

Figure 5b shows the XRD pattern of PVA polymer crosslinked with glutaraldehyde (GLU). As the amount of GLU increased, the peak intensity decreased, and the peak became broadened. Additionally, a peak shift from 2θ = 19.9° to 18.7° was noted, indicating a deformation of the (101) plane. These results suggest that the crystallinity of the polymer decreased as the degree of random crosslinking increased with increasing GLU content [30,33]. The XRD patterns of PVA-PCD polymers with 20 and 40 wt% PCD are shown in Figure 5c,d, with GLU contents from 1 to 30 wt%. Similar to the trend of crosslinked PVA polymers, the (101) plane peak at 2θ = 19.5° decreased in intensity and broadened with increasing GLU content, indicating a reduction in crystallinity due to crosslinking [32,33].

The variation in the crystallinity index (CI) of the PVA and PCD-PVA biopolymers with different GLU contents is summarized in Figure S3. For PVA only, the CI gradually decreased as the GLU content increased but remained relatively stable at 29–22%, with GLU >10 wt%. This trend indicates that a low content of GLU (<10 wt%) significantly affected the crystallinity of PVA but did not affect it further at high contents (>10 wt%). When PCD of 20 wt% was added (PVA(80)-PCD(20)), the CI remained almost unchanged beyond 2 wt% GLU, suggesting that GLU primarily facilitates crosslinking with PCD rather than PVA. For higher PCD contents (PVA(60)-PCD(40)), the CI continued to decrease with increasing GLU content, indicating a more uniform distribution of GLU to PVA and PCD. Interestingly, the decrease in the crystallinity index of the PCD-PVA biopolymer was less pronounced compared to that of crosslinked PVA alone. This phenomenon might be attributed to the smaller molecular size of PCD compared to PVA, allowing PCD to disperse into the amorphous regions of PVA and reducing its interaction with the more ordered PVA crystalline structures.

### 2.3. TGA

**Figure 6:**
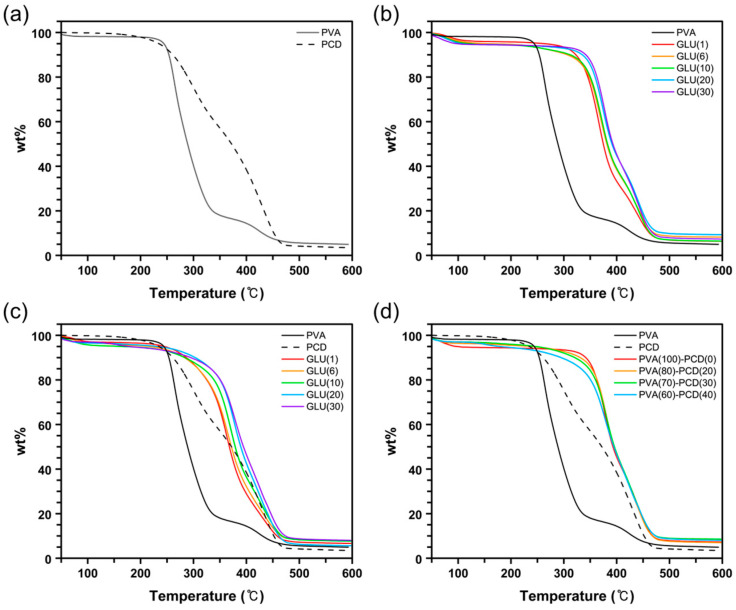
TGA curves for (**a**) base materials, (**b**) PVA polymers, (**c**) PVA(60)-PCD(40) biopolymers, and (**d**) PVA-PCD/GLU(30) biopolymers.

We performed a TGA analysis to investigate the thermal decomposition properties of PVA and polyols derived from cardanol (PCD) used in the fabrication of biodegradable polymers. The weight loss curves for PVA and PCD are presented in Figure 6a. The thermal degradation of PVA can be divided into three stages. The first stage occurs between 25 and 230 °C, at which a ~3 wt% loss is attributed to the evaporation of moisture adsorbed within the PVA. The second stage, from 240 to 350 °C, involved the dehydration of the PVA main chain, as well as the degradation of both the main and side chains, resulting in ~81 wt% of loss. Further decomposition of residual polyenes continued up to 480 °C, with a weight loss of ~9.5%, leaving ~5 wt% of carbonaceous residue below 500 °C. These findings show typical thermal degradation characteristics of PVA reported in the literature [34,35,36].

For PCD (Figure 6a), the initial weight loss of ~2 wt% was observed between 25 and 200 °C and was attributed to the evaporation of moisture and residual solvent during the synthesis. The second stage, from 200 to 470 °C, involved the degradation of the alkyl chains of cardanol, resulting in the largest weight loss of 89%. A notable inflection point was observed at 350 °C and was likely due to the simultaneous degradation of alkyl chains and the formation of oligomers through the polymerization of double bonds within the alkyl chains. The complete decomposition into gaseous volatile compounds occurred up to 470 °C, leaving ~3.5% of carbonaceous residue at 500 °C [37].

When PVA was crosslinked with GLU, the thermal properties of PVA were further investigated using TGA, with the results shown in Figure 6b. The temperature at 10% of weight loss and weight loss of each temperature were summarized in Table S3. The overall weight loss pattern was similar to that of pure PVA. However, as the GLU content increased, the onset temperature for the degradation of the main and side chains in the second stage shifted significantly from 240 °C for pure PVA to 300–340 °C, with the endpoint also increasing from 350 °C to 400 °C. Additionally, the final residue weight increased to a maximum of 10% with higher GLU content, indicating that the increased crosslink density enhanced the thermal stability of crosslinked PVA polymers [38].

To evaluate the crosslinking degree and thermal stability of the PVA-PCD biopolymers, TGA was performed on a PVA(60)-PCD(40) sample with varying GLU contents, as presented in Figure 6c. The temperature at 10% of weight loss and weight loss of each temperature section were summarized in Table S4. Up to 265 °C, the weight loss was ~8%, which was primarily due to the loss of weakly absorbed volatile substances, such as water. The overall pattern was similar to that of the PVA polymer, but in the second stage, in which rapid weight loss occurs between 250 and 490 °C, the decomposition temperature increased more significantly with higher crosslinker content, and the extent of this increase was greater compared to the PVA polymer. This result suggests that GLU effectively enhanced the thermal stability of the PVA-PCD biopolymers. The main factor that determines the crosslinking on the fabrication of PVA-PCD biopolymers is aldehyde groups of GLU reacting with the hydroxyl groups of PVA or PCD rather than hydroxyl groups of PVA or PCD reacting between PVA and PCD. Thus, this suggests that the functional groups of monomers are a more critical factor than the molecular sizes of PVA and PCD in the fabrication of PVA-PCD biopolymers via crosslinking. The final residue weight above 500 °C was ~9 wt%, which was lower than that of pure PVA but higher than that of pure PCD.

Figure 6d shows the TGA results for PVA-PCD biopolymers with a fixed GLU content of 30 wt% to study the effect of PCD contents on biopolymer’s thermal stability. The temperature at 10% of weight loss and weight loss of each temperature were summarized in Table S5. Similar to previous results, the following three distinct stages were observed: 25–240 °C for the loss of volatile substances, 290–480 °C for the decomposition of side chains and the polymer backbone, and above 500 °C for carbonization, leaving ~8% of residue. Notably, the weight loss at relatively low temperatures (90–393 °C) increased, and the onset of weight loss shifted from 300 °C to 250 °C as the PCD content increased. These results originate from increased volatilization of residual solvents used in the PCD polyol preparation. However, above 393 °C, the thermal degradation patterns were nearly identical, regardless of PCD content, indicating that PVA and PCD were uniformly crosslinked by GLU, resulting in a similar thermal degradation mechanism. Overall, the inclusion of PCD in crosslinked PVA slightly improved thermal stability compared to pure PVA, but further higher PCD contents had minimal impact. This suggests that the uniform dispersion of PCD polyol and GLU within the larger PVA molecules achieved effective crosslinking.

In conclusion, the increase in GLU content in the fabrication of polymers reduced weight loss at the same temperature, thereby enhancing thermal stability due to crosslinking. The increase in PCD content primarily affected the second degradation stage, confirming successful crosslinking between PVA and PCD. Thus, the GLU content is the most critical factor for improving the thermal stability of PVA-PCD biopolymers.

### 2.4. Swelling Test

**Figure 7:**
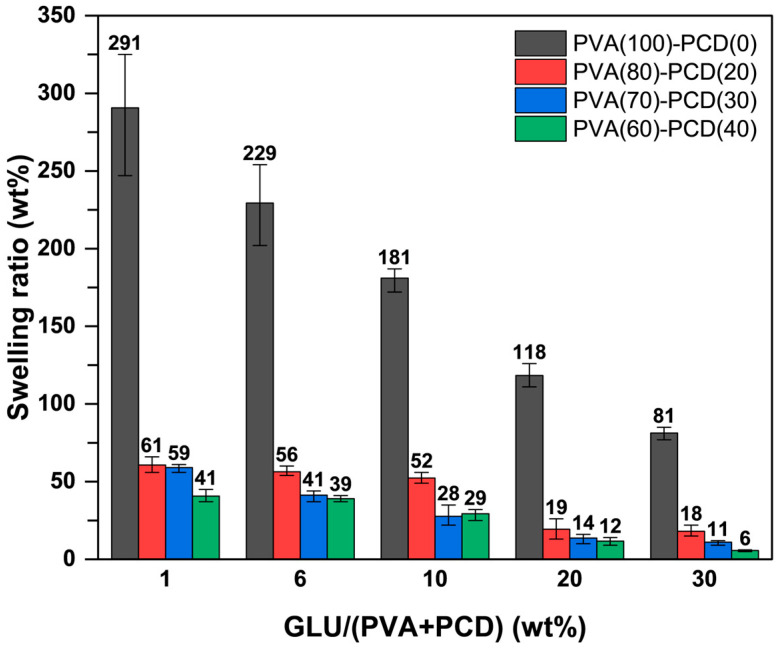
Swelling ratio of PVA-PCD biopolymers in PBS.

Due to their biodegradability and biocompatibility, biopolymers can be utilized in various applications, such as drug delivery, controlled-release fertilizers, antimicrobial films, and 3D-printed hydrogels. A key factor for these applications is the swelling performance of the biopolymer in aqueous solutions. Therefore, in this study, the swelling ratio of the synthesized PVA-PCD biopolymers was measured as described in the experimental methods shown in Figure 7.

For the PVA polymer, increasing the GLU content from 1 wt% to 30 wt% resulted in a significant decrease in the swelling ratio from 291% to 81%, which was a ~0.28 times reduction. This reduction is attributed to the decreased hydrophilicity due to the reduction of hydroxyl groups in the PVA and the increased crosslink density, which reduces water penetration into the hydrogel [26,39].

In the case of PVA-PCD biopolymers, the swelling ratio was lower compared to the PVA polymer. Specifically, PVA(80)-PCD(20) with a 20 wt% PCD content exhibited a decrease in swelling ratio from 61% to 18% as the GLU content increased, representing 0.30 times reduction. For PVA(60)-PCD(40), the swelling ratio decreased from 41% to 6%, for a 0.15 times reduction. Although higher PCD content in the PVA-PCD biopolymer generally decreased the swelling ratio more significantly, the trend was not directly correlated with the PCD content percentage.

Once PCD loses its hydroxyl groups after the reaction, it retains the phenolic lipid structure inherent to cardanol, resulting in hydrophobic properties. Thus, biopolymers formed with PCD polyol additions and crosslinking exhibit lower swelling ratios compared to PVA alone. Overall, the reduction in the swelling ratio is more strongly influenced by the PVA content than the PCD content. The reaction between the hydroxyl groups of PVA and PCD with GLU aldehyde forms acetal groups, leading to crosslinking, which reduces the hydrophilicity and promotes the formation of a hydrophobic crosslinked network. PVA-PCD biopolymers, with increased crosslinking, have lower swelling properties. By adjusting the crosslinker and PCD polyol content, the swelling properties of PVA polymers and PVA-PCD biopolymers can be tailored for various applications, giving them versatile uses.

## 3. Materials and Methods

### 3.1. Materials

Cardanol (CD) was provided by Chemifolio Co., Ltd. (Busan, Republic of Korea). Poly(vinyl) alcohol (99%, MW = 89,000–98,000) and glutaraldehyde (50 wt% solution) were purchased from Sigma-Aldrich (Burlington, MA, USA). Hydrochloric acid (35.0–37.0%) was purchased from Samchun Chemicals (Seoul, Republic of Korea).

### 3.2. Preparation

#### 3.2.1. Synthesis of Polyols Derived from Cardanol

First, epoxidized cardanol (ECD) was synthesized according to a similar method reported in the literature [20]. The reaction was performed in a 1 L four-neck flask equipped with a stirrer, reflux cooler, thermometer, and liquid pump (JWTC 600, Jeniewell Co., Ltd. (Seoul, Republic of Korea)). Cardanol (200 g), toluene (99.5%, 240 mL), formic acid (88–91%, 24 mL), and p-toluene sulfonic acid (99%, 4.3 g) were charged and heated at 50 °C. Hydrogen peroxide (30%, 215 mL) was dropped using a liquid pump. The mixture was stirred at 65 °C for 5 h. After the reaction, the liquid was cooled at room temperature and neutralized with 5 wt% sodium hydrogen carbonate solution and distilled water, respectively. Then, the organic phase was dried with sodium sulfate anhydrous (98.5%). Finally, toluene was removed using a rotary evaporator (N-1100, EYELA, Co., Ltd., Seongnam-si, Republic of Korea), and epoxidized cardanol brown liquid was obtained.

For polyols derived from cardanol (PCD), epoxidized cardanol (200 g) and 11% sulfuric acid solution (200 mL) were mixed at 70 °C for 5 h and cooled at room temperature. After phase separation, ethyl acetate (99.5%, 200 mL) was added to the organic phase and stirred. The mixture was neutralized, dried, and distillated using the same process. Dark brown-colored products were obtained.

#### 3.2.2. Fabrication of Biopolymers

Biopolymer was synthesized in a 100 mL round bottom flask equipped with a stirrer, reflux cooler, and thermometer. Hydrochloric acid solution (pH 2) and PVA were stirred at 95 °C for 1 h. The liquid was cooled at 60 °C, and 10 wt% cardanol-based polyol (PCD) dissolved in ethanol was added. The concentration of the mixture was 10 wt%. The amounts of PCD were controlled up to 40 wt% with respect to the PVA weight (PVA:PCD weight ratio ranged from 100:0 to 60:40). After 30 min of stirring, the crosslinker, glutaraldehyde (GLU), was added to 1–30 wt% for PVA and polyol. The mixture was stirred for 30 min. The films were casted on PET film using a 250 μm film applicator (YBA-6, YOSHIMITSU (Kyoto, Japan)), and the gels were casted in a Petri dish. All biopolymer samples were dried at 30 °C overnight and named PVA(wt%)-PCD(wt%)/GLU(wt%).

### 3.3. Characterization

#### 3.3.1. FT-IR

Fourier-transform infrared spectroscopy (FT-IR) was used to characterize specific chemical groups. FT-IR spectra were recorded with an FT-IR spectrometer (FT-4100, JASCO International Co., Ltd. (Tokyo, Japan)) using the attenuated total reflection (ATR) method within the wavenumber range of 4000–600 cm^−1^ under a resolution of 4 cm^−1^ for a 2 mm/s scan rate.

#### 3.3.2. XRD

X-ray diffraction (XRD) was used to investigate the crystallinity index (CI). The measurement was performed using the X’Pert3-Powder instrument (Malvern PANalytical (Malvern, UK)) with Cu-Kα radiation (λ = 1.54 Å) operating at 40 eV and 30 mA and scanning from 10° to 80° at a speed of 2 °/min. The crystallinity index (CI) was calculated following the method proposed by Abral et al. [22]. The maximum intensity I_T_ at the main peak, corresponding to the (101) lattice plane diffraction at 2θ = 19.5°, and the minimum intensity I_A_ before the main peak’s growth were measured. The CI was then determined using Equation (1), as follows:

CI (%) = {(I_T_ − I_A_)/I_T_} × 100 (1)

#### 3.3.3. TGA

A thermo gravimetric analysis (TGA) was used to measure thermal characteristics. The samples were tested using a thermo analyzer (TGA/DSC1, Mettler-Toledo AG (Greifensee, Switzerland)). The analysis was programmed from 25 °C to 600 °C, with a 10 °C/min heating rate under 50 mL/min N_2_ atmosphere.

#### 3.3.4. Swelling Test

The swelling behavior of hydrogel was determined using the Japanese Industrial Standard (JIS) K 7223 method [40]. The samples were weighed and placed in a nylon tea bag. The tea bags were immersed in phosphate buffer saline at 25 °C for 24 h. After immersion, the tea bag was taken out, and the excess water was drained for 15 min. The swollen state hydrogel samples were weighed (W_s_) and dried at 40 °C for 48 h. The dry-state hydrogel samples were also weighed (W_d_). The swelling ratio was determined using Equation (2).

Swelling ratio (%) = {(W_s_ − W_d_)/W_d_} × 100 (2)

## 4. Conclusions

In this study, we fabricated biodegradable biopolymers with unique structures and properties by crosslinking petrochemical-based polyvinyl alcohol (PVA), which is known for its biodegradability, with cardanol derived from cashew nut shells. We performed multiple characterizations to investigate its chemical structures and thermal stabilities and evaluated the swelling performance to test the applicability of this biodegradable biopolymer in various practical applications.

Purified cardanol was converted into polyols (PCD) via epoxidation followed by hydroxylation. An FT-IR analysis confirmed that the unsaturated double bonds in cardanol formed epoxy groups, which were subsequently converted into hydroxyl groups in the presence of water under acid catalysis. By using glutaraldehyde (GLU) as the crosslinker, we fabricated PVA-PCD biopolymers. As the amount of crosslinker increased, the degree of crosslinking increased due to the reduction of hydroxyl groups and the formation of acetal groups. An XRD analysis further confirmed that the crystallinity of crosslinked polymers decreased due to random crosslinking. The crosslinked PVA exhibited significantly improved thermal stability compared to raw PVA, although it showed reduced swelling capacity. Unlike the effect of the crosslinker, the impact of the PVA/PCD ratio on thermal stability was minimal, with the thermal degradation behavior resembling that of the crosslinked PVA polymer.

This work explored various critical properties of biopolymers, such as crosslinking degree, thermal stability, and swelling properties, by incorporating cardanol-based polyols into traditional biodegradable petrochemical PVA. These findings suggest potential future applications of biopolymers by using abundant biomass as an eco-friendly, biocompatible material with biodegradability. Next, the antimicrobial properties of cardanol-derived polyol (PCD) can suggest the development of antimicrobial PVA-PCD biopolymers.

## Data Availability

Data are contained within the article and Supplementary Materials.

## Supplementary Materials

The following supporting information can be downloaded at https://www.mdpi.com/article/10.3390/molecules29204807/s1: Figure S1: FT-IR spectra of crosslinked (a) PVA(80)-PCD(20) and (b) PVA(70)-PCD(30) biopolymers; Figure S2: Ratio of GLU associated with (a) hydroxyl group, (b) acetal group, and (c) ether bridge; Figure S3: Crystallinity index (CI) of PVA polymer and PVA-PCD biopolymer calculated using XRD results. Table S1: Assignment of functional groups associated with major vibration bands in CD, ECD, and PCD; Table S2: Assignment of functional groups associated with major vibration bands in PVA polymer and PVA-PCD biopolymer; Table S3: TGA results of crosslinked PVA polymer; Table S4: TGA results of PVA(60)-PCD(40) biopolymer; Table S5: TGA results of PVA-PCD/GLU(30) biopolymer.
