# Supplementary material for: Fabrication and Characterization of Biopolymers Using Polyvinyl Alcohol and Cardanol-Based Polyols
Source: Molecules. 2024 Oct 11;29(20):4807. doi: 10.3390/molecules29204807 (PMC11510699; doi:10.3390/molecules29204807)
Supplement: Supplementary file 1 [file molecules-29-04807-s001.zip › molecules-3230647-supplementary.pdf]

Supplementary files

Fabrication and Characterization of Biopolymers Using Polyvinyl Alcohol and Cardanol-Based Polyols

Da Hae Lee[a], Yun Ha Song[a], Hee Ju Ahn[a], Jaekyoung Lee[a,]*, Hee Chul Woo [a,]*,

[a]*Department of Chemical Engineering, Pukyong National University, 45 Yongso-ro, Nam-gu, Busan 48513, Korea*

* *Corresponding authors*

Tel.: +82 51 629 6433; fax: +82 51 629 7487

E-mail addresses: leejk46@pknu.ac.kr, woohc@pknu.ac.kr

Table S1. Assignment of functional groups associated with major vibration bands in CD, ECD, PCD

Functional group	Wavenumber (cm^{-1})	Ref.
O-H stretching at hydrogen bond system	3200–3500	[1,2]
C=C stretching from alkenes (unsaturated alkyl chain)	3010, 1611	[3]
C-H stretching from aliphatic chain	2925, 2853	[4]
C=O stretching (carboxyl, ester)	1723	[4,5]
C=C stretching of aromatic ring	1589, 1487	[1]
In-plane bending of C-H in alkyl chain	1456	[4]
In-plane bending of C-O-H in phenolic hydroxyl group	1350	[1]
C-O stretching between phenolic hydroxyl and aromatic ring	1263	[1]
C-O-C stretching from oxirane ring and ether bridges	1222	[6]
C-O stretching associated to hydroxyl end	1035–1067	[7]
Out-of-plane bending of C-H in methylene group	884, 911, 692	[8]
C-O-C stretching of epoxy group	824	[8]
Out-of-plane bending of C-H in aromatic ring	773, 692	[1]

Table S2. Assignment of functional groups associated with major vibration bands in PVA polymer and PVA-PCD biopolymer

Functional group	Wavenumber (cm^{-1})	Ref.
O-H stretching at hydrogen bonding	3267–3383	[1,2,9]
C-H stretching of aliphatic chain (from PVA and PCD)	2850–2935	[4,10,11]
C-H stretching from methylene groups	2933–2939	[4,10,11]
C=O stretching (free -CHO of GLU, carboxyl, ester, ketone)	1705–1715	[12,13]
C-H bending in alkyl chain	1417	[14]
Ehter linkages of acetal bridges	991–1007	[15]
C-H bending vibration in benzene ring	877–882, 744–763, 688–695	[1,8]
C-H bending vibration from PVA	778–783, 828–838	[9]

Table S3. TGA results of crosslinked PVA polymer

Sample	Temperature at 10% weight loss (°C)	Residual weight (wt%)			
		200 °C	300 °C	400 °C	500 °C
GLU(1)	322.9	95.8	93.5	33.3	6.9
GLU(6)	306.6	94.7	90.5	38.5	8.7
GLU(10)	312.0	94.3	90.9	39.1	7.0
GLU(20)	334.9	94.4	92.9	44.9	9.8
GLU(30)	341.6	94.3	93.5	45.4	7.9

Table S4. TGA results of PVA(60)-PCD(40) biopolymer

Sample	Temperature at 10% weight loss (℃)	Residual weight (wt%)			
		200 ℃	300 ℃	400 ℃	500 ℃
GLU(1)	286.8	96.4	87.2	29.0	7.4
GLU(6)	283.1	95.3	87.0	32.2	8.7
GLU(10)	283.7	94.6	87.9	36.5	8.3
GLU(20)	303.3	95.6	90.4	42.6	6.3
GLU(30)	295.0	94.5	89.5	46.5	8.7

Table S5. TGA results of PVA-PCD/GLU(30) biopolymer

**Sample**	**Temperature at 10% weight loss (℃)**	**Residual weight (wt%)**			
		**200 ℃**	**300 ℃**	**400 ℃**	**500 ℃**
PVA(100)-PCD(0)	341.6	94.3	93.5	45.4	7.9
PVA(80)-PCD(20)	331.2	95.5	92.8	46.6	7.6
PVA(70)-PCD(30)	320.9	96.0	92.1	47.1	8.9
PVA(60)-PCD(40)	295.0	94.5	89.5	46.5	8.7

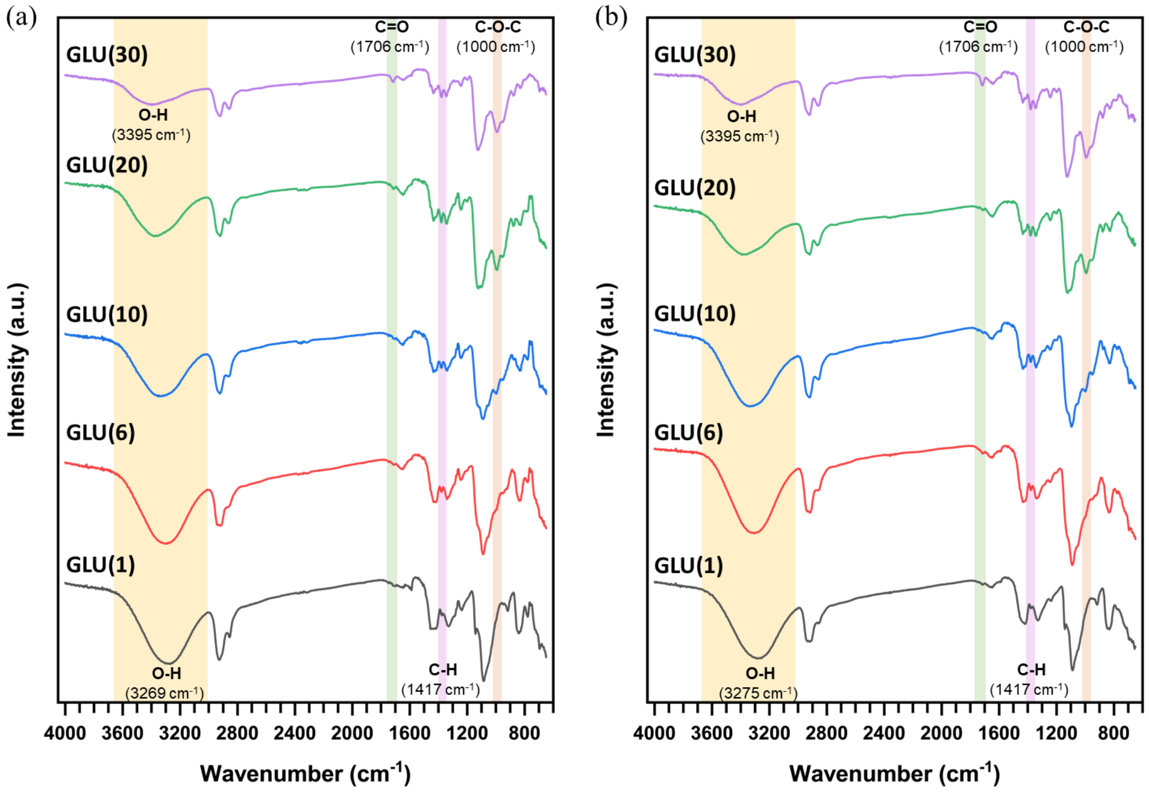

Figure S1. FT-IR spectra of crosslinked (a) PVA(80)-PCD(20) and (b) PVA(70)-PCD(30) biopolymers

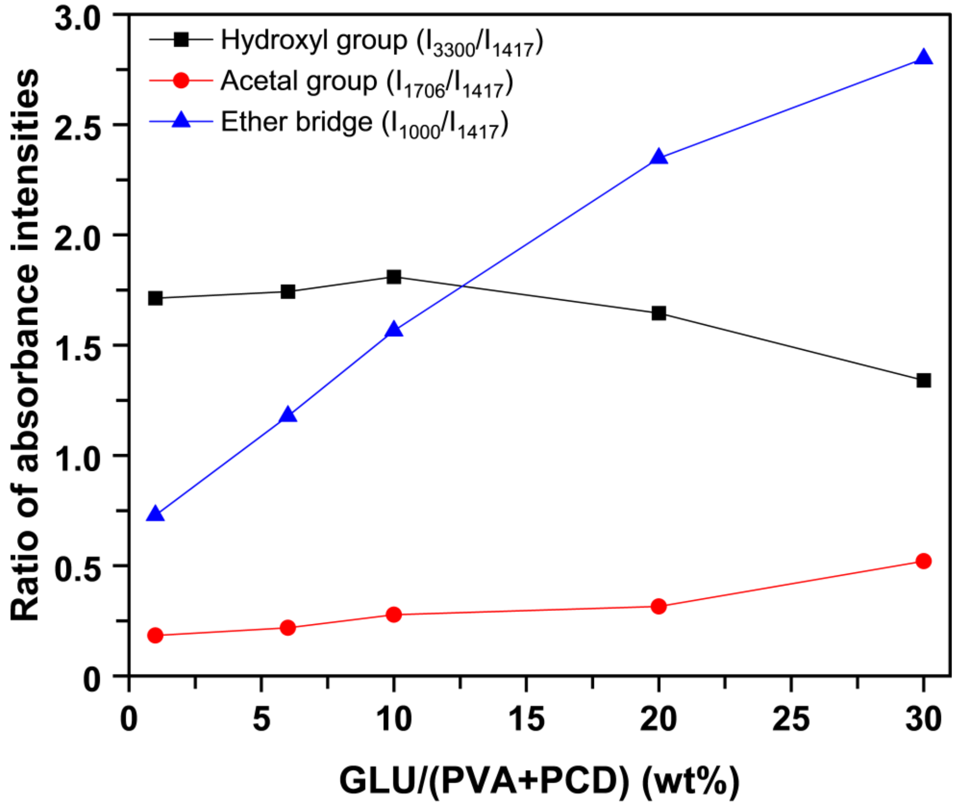

Figure S2. Ratio GLU associated with (a) Hydroxyl group (I_{3300}/I_{1417}) ,(b) acetal group (I_{1706}/I_{1417}) and (c) ether bridge (I_{1000}/I_{1417}).

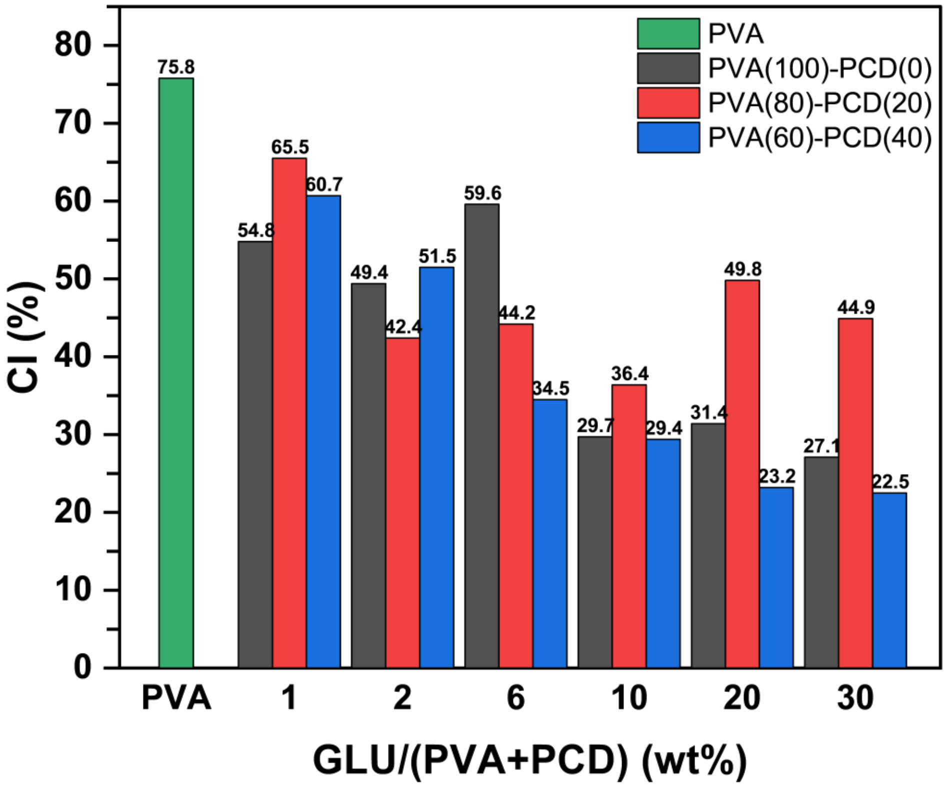

Figure S3. Crystallinity index (CI) of PVA polymer and PVA-PCD biopolymer calculated by XRD results.

References

1. Li, S.; Yang, X.; Huang, K.; Li, M.; Xia, J. Design, Preparation and Properties of Novel Renewable Uv-Curable Copolymers Based on Cardanol and Dimer Fatty Acids. *Prog. Org. Coat.* **2014**, *77*, 388-394.

2. Shukla, P.; Srivastava, D. Reaction Kinetics of Esterification of Phenol-Cardanol Based Epoxidized Novolac Resins and Methacrylic Acid. *Int. J. Plast. Technol.* **2014**, *18*, 1-15.

3. Fouquet, T.; Fetzer, L.; Mertz, G.; Puchot, L.; Verge, P. Photoageing of Cardanol: Characterization, Circumvention by Side Chain Methoxylation and Application for Photocrosslinkable Polymers. *RSC Adv.* **2015**, *5*, 54899-54912.

4. Hu, Y.; Shang, Q.; Bo, C.; Jia, P.; Feng, G.; Zhang, F.; Liu, C.; Zhou, Y. Synthesis and Properties of Uv-Curable Polyfunctional Polyurethane Acrylate Resins from Cardanol. *ACS Omega* **2019**, *4*, 12505-12511.

5. Sienkiewicz, A.M.; Czub, P. The Unique Activity of Catalyst in the Epoxidation of Soybean Oil and Following Reaction of Epoxidized Product with Bisphenol A. *Ind. Crop. Prod.* **2016**, *83*, 755-773.

6. Yang, F.; Yu, H.; Deng, Y.; Xu, X. Synthesis and Characterization of Different Soybean Oil-Based Polyols with Fatty Alcohol and Aromatic Alcohol. *e-Polymers* **2021**, *21*, 491-499.

7. Lopera-Valle, A.; Elias, A. Amine Responsive Poly(Lactic Acid) (PLA) and Succinic Anhydride (SAh) Graft-Polymer: Synthesis and Characterization. *Polymers* **2019**, *11*, 1466.

8. Liu, Z.; Chen, J.; Knothe, G.; Nie, X.; Jiang, J. Synthesis of Epoxidized Cardanol and Its Antioxidative Properties for Vegetable Oils and Biodiesel. *ACS Sustain. Chem. Eng.* **2016**, *4*, 901-906.

9. Abral, H.; Atmajaya, A.; Mahardika, M.; Hafizulhaq, F.; Kadriadi; Handayani, D.; Sapuan, S.M.; Ilyas, R.A. Effect of Ultrasonication Duration of Polyvinyl Alcohol (PVA) Gel on Characterizations of PVA Film. *J. Mater. Res. Technol.* **2020**, *9*, 2477-2486.

10. Wang, H.; Zhang, R.; Zhang, H.; Jiang, S.; Liu, H.; Sun, M.; Jiang, S. Kinetics and Functional Effectiveness of Nisin Loaded Antimicrobial Packaging Film Based on Chitosan/Poly(Vinyl Alcohol). *Carbohydr. Polym.* **2015**, *127*, 64-71.

11. Xu, Y.; Xu, Y.; Sun, C.; Zou, L.; He, J. The Preparation and Characterization of Plasticized PVA Fibres by a Novel Glycerol/Pseudo Ionic Liquids System with Melt Spinning Method. *Eur. Polym. J.* **2020**, *133*, 109768.

12. Mansur, H.S.; Sadahira, C.M.; Souza, A.N.; Mansur, A.A.P. Ftir Spectroscopy Characterization of Poly (Vinyl Alcohol) Hydrogel with Different Hydrolysis Degree and Chemically Crosslinked with Glutaraldehyde. *Mater. Sci. Eng. C* **2008**, *28*, 539-548.

13. Rudra, R.; Kumar, V.; Kundu, P.P. Acid Catalysed Cross-Linking of Poly Vinyl Alcohol (PVA) by Glutaraldehyde: Effect of Crosslink Density on the Characteristics of PVA

Membranes Used in Single Chambered Microbial Fuel Cells. *RSC Adv.* **2015**, *5*, 83436-83447.

14. Abral, H.; Hartono, A.; Hafizulhaq, F.; Handayani, D.; Sugiarti, E.; Pradipta, O. Characterization of PVA/Cassava Starch Biocomposites Fabricated with and without Sonication Using Bacterial Cellulose Fiber Loadings. *Carbohydr. Polym.* **2019**, *206*, 593-601.

15. Pandit, A.H.; Mazumdar, N.; Imtiyaz, K.; Rizvi, M.M.A.; Ahmad, S. Periodate-Modified Gum Arabic Cross-Linked PVA Hydrogels: A Promising Approach toward Photoprotection and Sustained Delivery of Folic Acid. *ACS Omega* **2019**, *4*, 16026-16036.
